# FOLFIRI plus panitumumab in the treatment of wild-type KRAS and wild-type NRAS metastatic colorectal cancer

**DOI:** 10.1186/s12957-018-1359-9

**Published:** 2018-03-27

**Authors:** Caglayan Geredeli, Nurgul Yasar

**Affiliations:** Department of Medical Oncology, Okmeydani Training and Research Hospital, Sisli, Istanbul, Turkey

**Keywords:** FOLFIRI, Panitumumab, Colorectal cancer

## Abstract

**Background:**

The aim of this study was to investigate the efficacy and safety of first-line panitumumab plus folinic acid, 5-fluorouracil and irinotecan (FOLFIRI) in patients with wild-type KRAS and wild-type NRAS metastatic colorectal cancer (mCRC).

**Methods:**

Patients with wild-type KRAS and wild-type NRAS mCRC presenting to the medical oncology department of the Okmeydani Training and Research Hospital in Istanbul, Turkey, between April 2014 and January 2018 were enrolled in this study.

**Results:**

A total of 64 patients (35 males and 29 females) with a median age of 59 (35–81) years old were enrolled. The median follow-up was 18.9 months, and the median progression-free survival was 13 months. The median overall survival (OS) was 26 months in the patients with wild-type KRAS and wild-type NRAS mCRC. It was 90.4% for the 6-month OS, 79.5% for the 1-year OS, 53.7% for the 2-year OS and 31.1% for the 3-year OS. The median OS of the patients who underwent metastasectomies was 40 [95% confidence interval (CI) = 19.9–60.1] months, and the median OS of the patients without metastasectomies was 22 (95% CI = 17.7–26.4) months. There was a statistically significant difference between these (*P* = 0.007).

**Conclusion:**

The first-line FOLFIRI plus panitumumab was associated with favourable efficacy in the patients with wild-type KRAS and wild-type NRAS mCRC, and it was well tolerated. The removal of the metastases that became resectable after chemotherapy further prolonged the patients’ survival.

**Trial registration:**

Retrospectively registered: 33886

## Background

Folinic acid, infusional 5-fluorouracil and irinotecan (FOLFIRI) is a regimen recommended for use as both a first-line and second-line treatment for metastatic colorectal cancer (mCRC) [1]. Epidermal growth factor receptor (EGFR) inhibitors (panitumumab and cetuximab) can be used in combination with folinic acid, infusional 5-fluorouracil and oxaliplatin (FOLFOX) and FOLFIRI regimens in the treatment of wild-type KRAS (Kirsten rat sarcoma viral oncogene homologue) and wild-type NRAS mCRC patients [2]. The combination of an EGFR inhibitor plus the FOLFOX or FOLFIRI regimen has improved survival in first- and second-line treatments [2–7]. Initially, the combination of panitumumab plus FOLFOX was used in the treatment of mCRC patients, and it was found to be effective without impairing the quality of life [8, 9]. For the second-line treatment of wild-type RAS (both wild-type KRAS and wild-type NRAS) mCRC patients, the panitumumab plus FOLFIRI regimen was found to be superior to the FOLFIRI regimen alone with regard to the disease-free survival [10]. In a few studies, the FOLFIRI plus panitumumab regimen was found to be effective and safe for the first-line treatment of wild-type KRAS mCRC patients [11–14]. Here, we would like to present our single-centre experience regarding the efficacy and safety of the FOLFIRI plus panitumumab regimen as the first-line treatment in wild-type KRAS and wild-type NRAS mCRC patients.

## Methods

Wild-type KRAS and wild-type NRAS mCRC patients followed up at the medical oncology department of the Okmeydani Training and Research Hospital in Istanbul, Turkey, between April 2014 and January 2018 were enrolled in this retrospective study. After examining their files, those patients with histologically confirmed colon cancer diagnoses, radiologically confirmed metastases, genetically established KRAS and NRAS statuses and performance statuses of 0–2 on the Eastern Cooperative Oncology Group (ECOG) scale were included in this study. The KRAS and NRAS analyses were conducted using a real-time polymerase chain reaction (PCR) on the DNA extracted from fixed tumour sections. We performed a two-round nested PCR for the amplification of exons 1, 2 and 3 of the KRAS and NRAS genes harbouring codons 12, 13, 59, 61, 117 and 146. This was followed by a multiplex mini-sequencing reaction for the detection of potential mutations.

As a first-line treatment, panitumumab (6 mg/kg), FOLFIRI (irinotecan (180 mg/m^2^) and leucovorin (400 mg/m^2^), followed by a 400 mg/m^2^ bolus of 5-fluorouracil and a 2400–3000 mg/m^2^ continuous infusion regimen, was administered every 14 days for 6 cycles. In addition, 100 mg of doxycycline (twice daily) and corticosteroid creams were used during the chemotherapy to reduce the dermatological side effects of the panitumumab. This was a prophylactic treatment started after the chemotherapy treatment actually caused skin toxicity.

The patients were evaluated using radiological imaging methods [computed tomography (CT)] every 8 weeks according to the Response Evaluation Criteria in Solid Tumours (RECIST1.1) criteria. The objective response rate (ORR), progression-free survival (PFS) and overall survival (OS) durations were calculated. The safety was evaluated in terms of the incidence and severity of adverse events (AEs), using the National Cancer Institute common toxicity criteria version 3.0.

The Statistical Package for the Social Sciences (SPSS) version 15.0 for Windows was used for the statistical analysis. The descriptive statistics were expressed as the mean, standard deviation, minimum, maximum and median for the numerical variables, and they were expressed as numbers and percentages for the categorical variables. If the numerical variables of two independent groups were not normally distributed, they were analysed using a Mann-Whitney *U* test. A chi-squared test was used for the comparisons of the ratios in the groups, and a Monte Carlo simulation was applied when the conditions were not met. The survival analyses were conducted with a Kaplan-Meier analysis, and the statistical significance level of alpha was accepted as *p* < 0.05.

## Results

A total of 64 patients were enrolled in this study. The median age was 59 years old (range = 35–81 years): 35 patients were males and 29 were females. From a localization point of view, 56 patients had left colon tumours (87.5%), and 8 patients had right colon tumours (12.5%). While 45 (70.3%) patients were diagnosed with synchronous metastases, and 19 (29.7%) patients had metachronous metastases. During the follow-up period, 46 (71.9%) had liver metastases, 7 (10.9%) had lung metastases, 4 (6.3%) had bone metastases, 4 (6.3%) had peritoneal metastases and 3 (4.7%) had remote lymph node metastases (Table 1). The median follow-up duration was 18.9 months. Progression was seen in 50 (78.1%) patients, while 14 (21.9%) showed no progression. Thirty-three (51.6%) patients died during the follow-up (Table 1).

**Table 1:** Patient characteristics

Characteristics		*n*	Percentile (%)
Sex	Female	29	45.3
	Male	35	54.7
Pathology	Adenocarcinoma	61	95.3
	Mucinous	3	4.7
Grade	I	4	6.3
	II	57	89.1
	III	3	4.7
KRAS	Wild	64	100
NRAS	Wild	64	100
Biopsy location	Primary tumour	61	95.3
	Metastasis	3	4.7
Metastasis diagnosed	Synchronous	45	70.3
	Metachronous	19	29.7
Right colon	Total	8	12.5
	Ascending colon	5	7.8
	Transverse colon	3	4.7
Left colon	Total	56	87.5
	Rectum	21	32.8
	Sigmoid colon	33	51.6
	Descending colon	2	3.1
Metastasis location	Liver	46	71.9
	Lung	7	10.9
	Peritoneum	4	6.3
	Bone	4	6.3
	Remote lymph node	3	4.7
Metastasectomy	Yes	17	26.5
	No	47	73.5
Second treatment	Yes	40	62.5
	No	24	37.5
Progression	Yes	50	78.1
	No	14	21.9
Death	Yes	33	51.5
	No	31	48.5

**Fig. 1:**
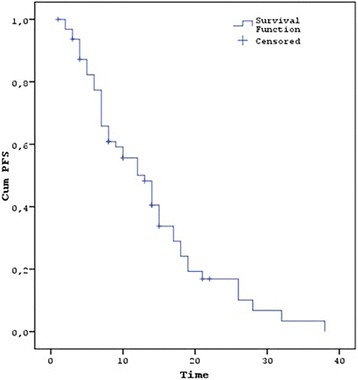
PFS of patients with wild-type KRAS and wild-type NRAS mCRC

**Fig. 2:**
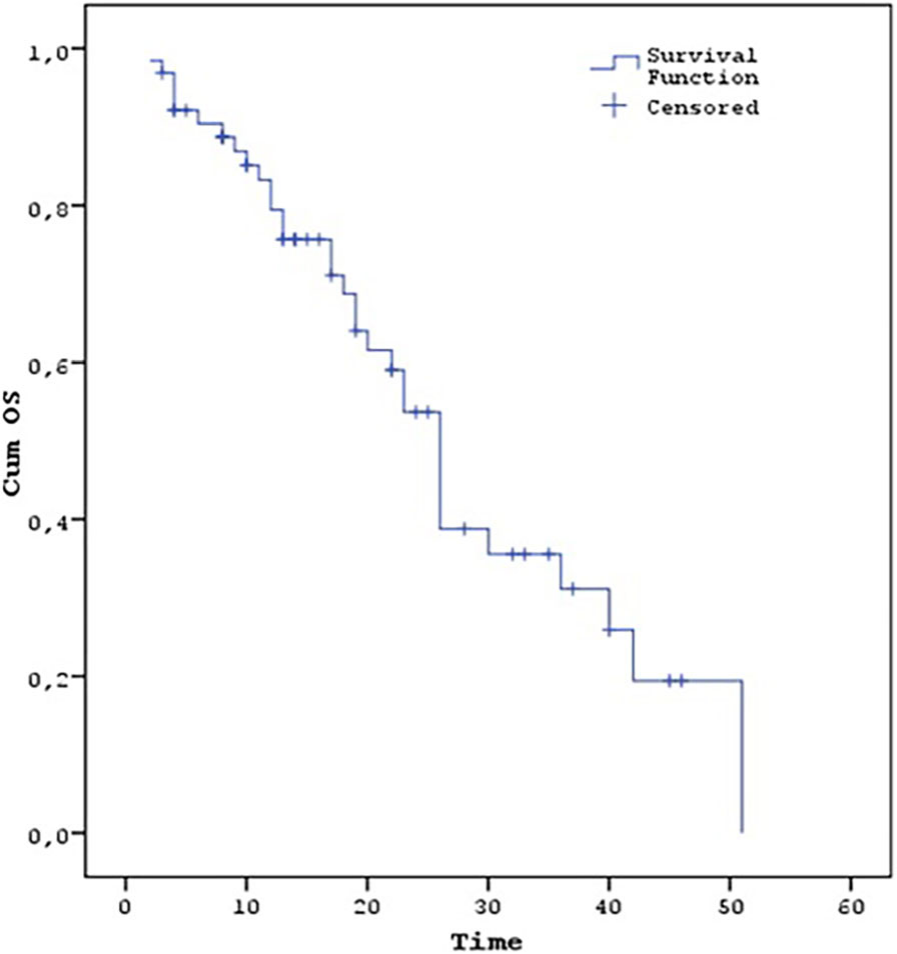
OS of patients with wild-type KRAS and wild-type NRAS mCRC

**Fig. 3:**
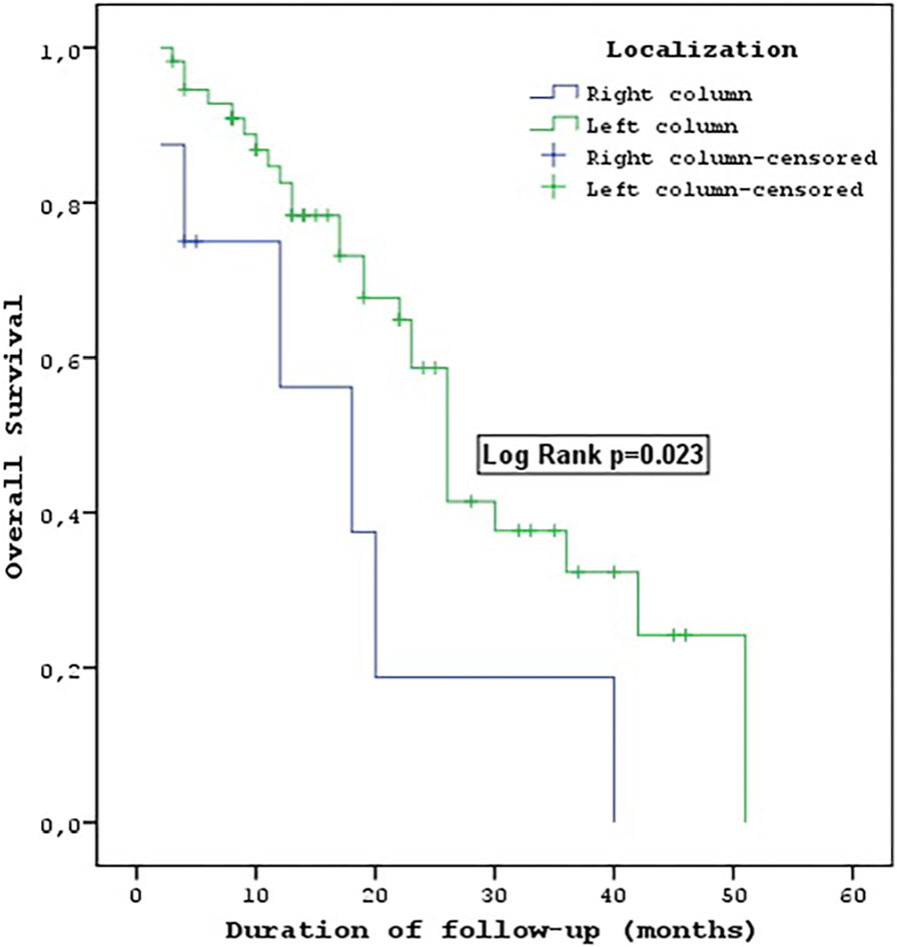
OS tumour location patients with wild-type KRAS and wild-type NRAS mCRC

After 8 weeks of therapy, the evaluation of the response rates indicated that a complete response (CR) was achieved in 3 patients (4.7%), 50 (78.1%) showed a partial response (PR), 5 had stable disease (SD) (7.8%) and 6 (9.4%) had progressive disease (PD) (Table 2). The median PFS of the wild-type KRAS and wild-type NRAS mCRC patients was 13 [95% confidence interval (CI) = 9.6–16.4] months (Fig. 1), with 77.3% 6-month, 50.1% 1-year, 16.9% 2-year and 3.4% 3-year survivals (Table 2). FOLFOX-bevacizumab was administered as second-line chemotherapy in 40 (62.5%) of the 50 (78.1%) patients with disease progression during the follow-up period of 18.9 months (Table 1). The median OS was 26 (95% CI = 23–29) months (Fig. 2), with 90.4% 6-month, 79.5% 1-year, 53.7% 2-year and 31.1% 3-year survivals (Table 2). The median PFS was 4 (95% CI = 1.5–6.5) months for the patients with right colon tumours. In contrast, it was 14 (95% CI = 10.8–17.2) months for the patients with left colon tumours, and the difference was statistically significant (*P* = 0.02). The median OS was 18 (95% CI = 5.3–30.7) months in the patients with right colon tumours and 26 (95% CI = 23.1–28.9) months in the patients with left colon tumours (Table 2) (Fig. 3). This difference was also statistically significant (*P* = 0.02).

**Table 2:** Response, PFS, OS and toxicity rate

Response to treatment	*n*	%	*p*
Complete response	3	4.7	
Partial response	50	78.1	
Stable disease	5	7.8	
Progressive disease	6	9.4	
Wild-type KRAS and NRAS PFS	Months or %	%95 CI (min-max)	
Median follow-up	18.9	2.0–51.0	
Median PFS	13	9.6–16.4	
6-months PFS	77.3		
1-year PFS	50.1		
2-year PFS	16.9		
3-year PFS	3.4		
Wild-type KRAS and NRAS OS			
Median OS	26	23.1–29.2	
6-months OS	90.4		
1-year OS	79.5		
2-year OS	53.7		
3-year OS	31.1		
Tumour location			
Right colon PFS	4	1.5–6.5	*0.02*
Left colon PFS	14	10.8–17.2	
Right colon OS	18	5.3–30.7	*0.02*
Left colon OS	26	23.1–28.9	
Metastasectomy			
Yes PFS	17	14.3–19.7	*0.02*
No PFS	8	5.6–10.4	
Yes OS	40	19.9–60.1	*0.007*
No OS	22	17.7–26.4	
Metastases diagnosed			
Synchronous PFS	12	7.0–17.0	0.28
Metachronous PFS	17	3.6–30.4	
Synchronous OS	23	16.9–29.1	0.21
Metachronous OS	26	21.7–30.3	
Toxicity	Grade (all)	Grade (1–2)	Grade (3–4)
Acne-like rash	%60.9	%54.6	%6.3
Diarrhoea	%36.2	%31.5	%4.7
Neutropenia	%34.7	%25.5	%9.2

*PFS* progression-free survival, *OS* overall survival

**Fig. 4:**
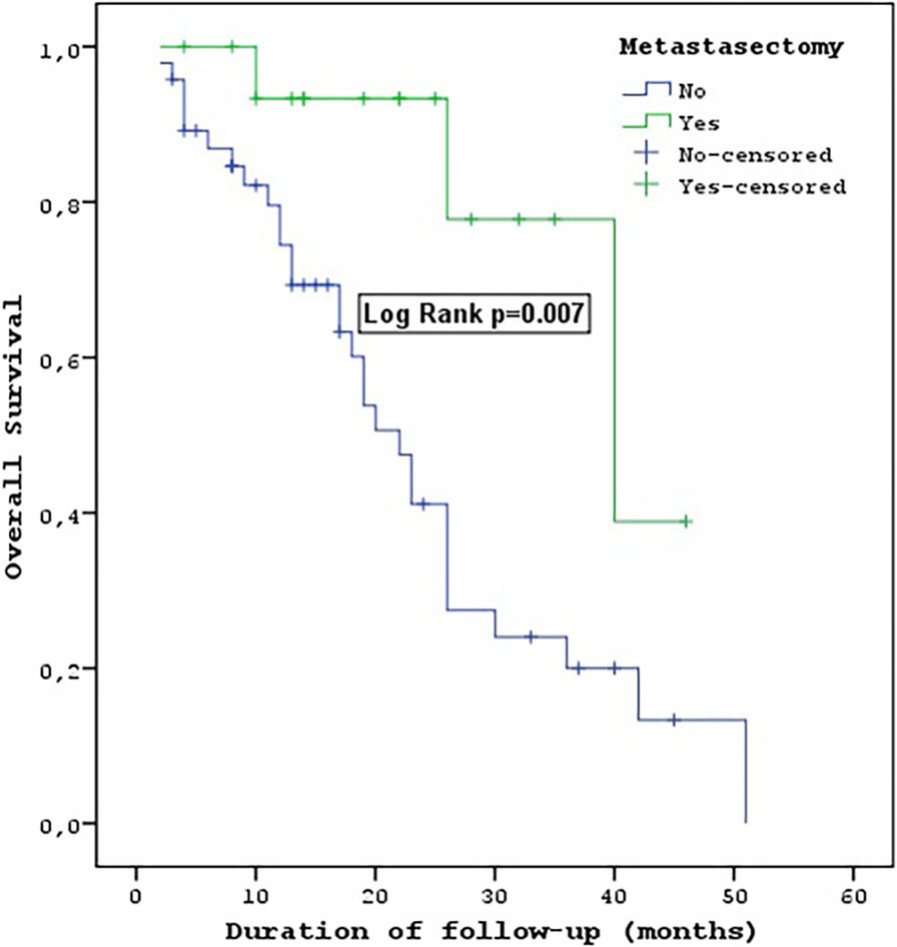
OS metastasectomy of patients with wild-type KRAS and wild-type NRAS mCRC

Of the patients, 17 (26.6%) underwent metastasectomies, and of these, 15 (13.4%) underwent metastasectomies for liver metastases and 2 (3.1%) underwent metastasectomies for lung metastases. The median PFS of the patients who underwent metastasectomies was 17 (95% CI = 14.3–19.7) months, and the median PFS of the patients without metastasectomies was 8 (95% CI = 5.6–10.4) months. The median PFS of the metastasectomy patients was statistically significantly longer (*P* = 0.02). The median OS of the patients who underwent metastasectomies was 40 (95% CI = 19.9–60.1) months, and the median OS of the patients without metastasectomies was 22 (95% CI = 17.7–26.4) months. There was a statistically significant difference between these values (*P* = 0.007) (Table 2) (Fig. 4).

The PFS was 12 (95% CI = 7–17) months in the patients with synchronous metastases, and the PFS was 17 (95% CI = 3.6–30.4) months in those with metachronous metastases. However, no statistical difference was found (*P* = 0.28). The OS was 26 (95% CI = 21.7–30.3) months in those with metachronous metastasis, and it was 23 (95% CI = 16.9–29.1) months in those with synchronous metastases. No statistical difference was found between them (*P* = 0.21) (Table 2).

The assessment of toxicity showed that the most frequently occurring symptoms of grade 1/2 toxicities were an acne-like rash (60.9%), diarrhoea (36.2%) and neutropenia (34.7%). Similarly, the most frequently occurring symptoms of grade 3/4 toxicities were diarrhoea (4.7%), an acne-like rash (6.3%) and neutropenia (9.2%) (Table 2).

## Discussion

In our study, we evaluated the effects of the combination of panitumumab, an anti-EGFR drug, and FOLFIRI chemotherapy on the patients’ survival. In the PRIME trial, after using panitumumab plus FOLFOX as the first-line treatment, the PFS was 10.1 months and the OS was 26 months in the wild-type RAS patients [15]. The 26-month OS outcomes of both the wild-type RAS mCRC patients in our study were found to be identical to those of the 26-month OS in the PRIME trial.

In the PEAK trial, after using the panitumumab plus FOLFOX as the first-line treatment in mCRC patients in that trial, the PFS was 13.0 months and the OS was 41.3 months in the wild-type RAS patients [8]. The 13-month PFS outcomes of wild-type RAS mCRC patients in our study were found to be identical to those of the 13-month PFS in the PEAK study. The 26-month OS outcomes of wild-type RAS mCRC patients in our study were found to be lower than those of the 41.3-month OS in the PEAK study.

In the CRYSTAL study that was conducted with cetuximab (another anti-EFGR drug), the PFS was 11.4 months and the OS was 28.4 months in the wild-type RAS patients who received FOLFIRI plus cetuximab [15]. Our findings of 13 months for the PFS and 26 months for the OS were similar to those of the 11.4-month PFS and 28.4-month OS in the CRYSTAL study results. Moreover, in the FIRE-3 trial, the PFS in the wild-type RAS mCRC patients was 10.4 months and the OS was 33.1 months [16]. Our 13-month PFS result was longer than the 10.4-month PFS result from the FIRE-3 trial, but the 26-month OS result was shorter than that of the 33.1-month OS from that trial. Finally, the CAPRI-GOIM trial used FOLFIRI plus cetuximab, and the median PFS was 11.1 months in the wild-type RAS mCRC patients [17].

In the first prospective phase 2 trial using a FOLFIRI plus panitumumab combination in wild-type KRAS patients, the time to progression (TTP) was reported as 11.2 months by Köhne et al. [10]. In a study by Karthaus et al., the PFS was 11.2 months in the wild-type KRAS patients [18]. The PFS was 13 months for the wild-type RAS patients in our study, showing that our results were better than those of Karthaus and Köhne et al.

In this study, even though our patient number was low, we also analysed the tumour localization rates, which have recently become a highly discussed topic. In the retrospective analyses indicating the tumour localization [19, 20], the PFS was 7.5–8.7 months (7.5–7.6–8.1–8.7) in the right colon patients and 10.7–14.6 months (10.7–11.1–12–14.6) in the left colon patients for those using the FOLFIRI plus cetuximab and FOLFOX plus panitumumab regimens in the PRIME study, FIRE-3 study, CRYSTAL study and PEAK study. Furthermore, in the PRIME study, PEAK study, FIRE 3 study and CRYSTAL study, the OS was 11.1–18.5 months (11.1–17.5–18.3–18.5) in the right colon patients and 28.7–43.4 months (28.7–30.3–38.3–43.4) in the left colon patients (21) [19, 20]. Out of the 64 patients in our study, only 8 had right colon tumours. The PFS was 4 months in the patients with right colon tumours and 14 months in those with left colon tumours. We found that OS was 18 months in the patients with right colon tumours and 26 months in those with left colon tumours. There was a statistically significant difference regarding the tumour localization (*p* = 0.02).

Although the patient groups and chemotherapy regimens differed, many studies have shown that patients with mCRC with limited metastases undergoing only liver metastasectomies may exhibit prolonged survival if the metastases become resectable after chemotherapy [21–29]. In the subgroup analyses of the Köhne et al. and CRYSTAL studies, in the wild-type RAS patients with liver-limited disease being treated with FOLFIRI plus cetuximab, the PFS was 14 months and the OS was 29.8 months [26]. In our study, the PFS was 17 months and the OS was 40 months in the patients who had liver metastases resected after FOLFIRI plus panitumumab chemotherapy. The removal of metastases that become resectable after chemotherapy can further prolong the patient’s survival.

In our retrospective study, the diarrhoea rate was 36.2% and that of an acne-like rash was 60.9% in all the grades. In the study by Köhne et al., the rate of diarrhoea was 23% and that of dermatological toxicity was 29% [11]. Although our rates seemed to be higher than the rates in Köhne et al.’s study, other studies have reported higher diarrhoea and dermatological toxicity rates [30]. In the PEAK study, serious AEs were observed in 7% of the wild-type KRAS mCRC patients using FOLFOX plus panitumumab [8]. In our study, 4.7% diarrhoea and 6.3% acne-like rash rates occurred as grade 3/4 toxicity symptoms. Our rates of serious AEs were found to be very similar to the results of the PEAK study. In our study, we observed that the diarrhoea and skin toxicity, which are the most common side effects of the FOLFIRI plus panitumumab regimen, could be easily managed with prophylactic precautions (100 mg of doxycycline twice per day, as well as antibiotic and corticosteroid creams) used after the chemotherapy-induced skin toxicity develops.

## Conclusions

The results of this study showed that a first-line panitumumab plus FOLFIRI treatment was associated with favourable efficacy in patients with wild-type RAS mCRC, and it was well tolerated. FOLFIRI plus panitumumab is much more effective in left colon tumours but is largely ineffective in right colon tumours. The removal of metastases that become resectable after chemotherapy can further prolong the patient’s survival.
